# Feasibility and Outcomes of Intravenous Lithotripsy as an Adjunct in Complex Transvenous Lead Extraction

**DOI:** 10.1111/jce.70325

**Published:** 2026-03-24

**Authors:** Mohamad Mdaihly, Joe Demian, Arshneel S. Kochar, David O. Martin, Aravinda Nanjundappa, Arwa Younis, Ali Dakik, Jaafar Ismail, Issam Motairek, Chadi Tabaja, Adele Watfa, Pasquale Santangeli, Ayman A. Hussein, Oussama M. Wazni, Thomas D. Callahan

**Affiliations:** ^1^ Department of Cardiovascular Medicine Cardiac Electrophysiology and Pacing Section, Cleveland Clinic Cleveland Ohio USA

**Keywords:** complications, intravascular lithotripsy (IVL), procedural success, transvenous lead extraction (TLE)

## Abstract

**Background:**

Transvenous lead extraction (TLE) of old leads is associated with increased procedural complexity and risk due to the development of calcified vascular adhesions. This may necessitate additional tools to free the leads. Shockwave intravenous lithotripsy (IVL), which employs acoustic pressure waves to fracture calcified lesions, has emerged as an adjunctive tool in this setting. However, data on its feasibility in TLE remains limited.

**Objective:**

To evaluate the feasibility of IVL as an adjunctive tool in complex TLE cases, focusing on the extraction tools utilized, fluoroscopy duration, success rates, and associated complications.

**Methods:**

From a prospectively maintained registry, we analyzed all patients undergoing IVL‐assisted TLE at our institution. These patients were matched to controls without IVL using propensity scores derived from baseline and procedural characteristics, including age, sex, infection indication, age of the oldest explanted lead, number of extracted leads, and number of extracted ICD leads. A matching ratio of 1:3 was applied to increase statistical power while maintaining covariate balance.

**Results:**

A total of 27 IVL‐assisted cases were matched to 81 controls. Baseline demographics and comorbidities were well balanced between groups. The mean number of extracted leads and mean age of the oldest extracted lead were comparable (2.44 ± 0.98 vs. 2.49 ± 0.90, *p* = 0.81; 15.35 ± 5.80 vs. 15.78 ± 8.24 years, *p* = 0.80). IVL patients demonstrated significantly higher utilization of laser sheaths, either alone (100% vs. 77.7%, *p* = 0.006) or combined with mechanical tools (44.4% vs. 18.5%, *p* = 0.008). Median fluoroscopy time was significantly longer in the IVL group (50.2 vs. 15.7 min, *p* < 0.001). Complete IVL application from the brachiocephalic vein to the superior vena cava was achieved in 20 patients, while 7 were limited by proximal venous stenosis. Procedural and clinical success rates, as well as major and minor complications, were not significantly different between groups.

**Conclusion:**

We report the largest series on the use of shockwave IVL during complex TLE cases, with significantly higher fluoroscopy and procedure durations. Despite favorable safety and efficacy outcomes, adjunctive IVL did not confer clear clinical benefit, and the complexity of lead removal remained evident.

## 1 Introduction

**FIGURE 1:**
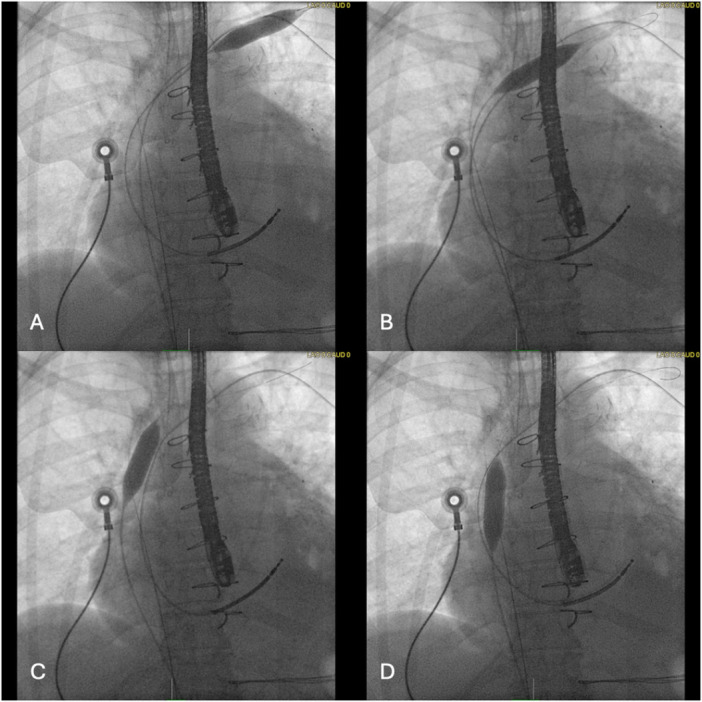
Lithotripsy treatment starting in the distal brachiocephalic vein (A), central brachiocephalic vein (B), brachiocephalic‐SVC junction(C), and SVC (D).

Transvenous lead extraction (TLE) of chronically implanted leads is often complicated by dense, calcified vascular adhesions that develop over long dwell time, increasing procedural complexity and complications [1]. Several extraction tools have been designed to assist in TLE, typically by cutting away at the adhesions. Intravenous lithotripsy (IVL) is a balloon‐based technology that employs acoustic pressure waves to fracture calcified vascular lesions [2]. IVL has recently been applied to break calcified adhesions around implanted leads to facilitate extraction (Figure 1). However, data on its feasibility and safety remain limited to a small case series and case reports [3, 4, 5].

We aimed to evaluate the feasibility of IVL as an adjunctive tool in complex TLE cases.

## 2 Methods

Using the prospectively maintained Extraction and Transvenous Removal: Advancing CIED Therapies (ExTRACT) registry, we identified all patients who underwent IVL‐assisted TLE at our institution. Patients were divided into two groups based on IVL use. Procedural success and complications were defined according to expert consensus statement [1]. Complete procedural success was defined as the removal of all targeted leads and lead material without disabling complications or death. Clinical success was defined as the extraction of all targeted leads or lead material, or retention of a small fragment (< 4 cm) without negatively impacting the procedural outcomes goals.

All TLE procedures were performed following our standardized institutional protocol [6]. IVL use was left to the operator's discretion, mainly in cases with long lead dwell times or when pre‐procedural imaging suggested significant calcification. A 30 mm balloon catheter was utilized, with 300 pulses delivered per procedure, except for the first case in which two balloons were used (total 600 pulses). Standard extraction sheaths and tools were then used to complete lead removal.

Statistical analyses were performed using SPSS Statistics. Descriptive statistics summarized baseline characteristics and outcomes. To reduce confounding, IVL patients were matched to controls using propensity scores derived from baseline and procedural variables, including age, sex, infection indication, age of the oldest explanted lead, number of extracted leads, and number of extracted ICD leads. Nearest neighbor matching without replacement was performed using a caliper width of 0.1 on the propensity score scale. A matching ratio of 1:3 was applied to increase statistical power while maintaining covariate balance. Covariate balance before and after matching was evaluated using standardized mean differences (SMDs), with values < 0.1 considered optimal and values < 0.2 considered acceptable. The study was approved by the Cleveland Clinic Institutional Review Board.

## 3 Results

A total of 27 patients who underwent lithotripsy were successfully matched to 81 controls. Baseline characteristics were well balanced between the groups (Table 1).

**TABLE 1:** Baseline demographics.

Characteristics	IVL (*n* = 27)	Conventional extraction (*n* = 81)	*p*‐value
Age (y)	58.85 ± 18.33	59.46 ± 15.12	0.87
Sex: male	19 (70.4)	58 (71.6)	1.000
EF	45.08 ± 12.0	44.34 ± 15.53	0.83
CABG	2 (7.4)	13 (16.0)	0.35
HTN	14 (51.9)	36 (44.4)	0.51
DM	8 (29.6)	16 (19.8)	0.30
CVD	0	0	
CAD	10 (37.0)	25 (30.9)	0.64
PAD	1 (3.7)	0	0.25
COPD	4 (14.8)	6 (7.4)	0.26

Abbreviations: CABG, coronary artery bypass grafting; CAD, coronary artery disease; COPD, chronic obstructive pulmonary disease; CVD, cerebrovascular disease; DM, diabetes mellitus; EF, ejection fraction; HTN, hypertension; PAD, peripheral artery disease.

The mean number of extracted leads was similar between the groups (2.44 ± 0.98 in the IVL group vs. 2.49 ± 0.90 in conventional groups, *p* = 0.81), as was the mean age of the oldest extracted lead (15.35 ± 5.80 years vs. 15.78 ± 8.24 years, respectively, *p* = 0.80). In the IVL group, manual traction alone was insufficient for successful extraction in any case (Table 2). IVL patients demonstrated significantly higher utilization of laser sheaths, either alone or combined with mechanical tools (Table 2). Median fluoroscopy times were significantly higher in the IVL group (50.2 vs. 15.7 min, *p* < 0.001).

**TABLE 2:** Procedural characteristics.

Variable	IVL (*n* = 27)	Conventional extraction (*n* = 81)	*p*‐value
Number of extracted leads	2.44 ± 0.98	2.49 ± 0.90	0.81
Oldest lead age (y)	15.35 ± 5.80	15.78 ± 8.24	0.80
Total lead age (y)	34.13 ± 17.78	33.70 ± 21.80	0.93
ICD system in place	20 (74.1)	57 (70.4)	0.81
Number of explanted ICD leads*	0.89 ± 0.75	0.98 ± 0.82	0.63
Infectious indication for extraction	15 (55.6)	43 (53.1)	1.000
Non‐infectious indication for extraction	12 (44.4)	38 (46.9)	1.000
Powered sheath use			
1‐ Mechanical	12 (44.4)	26 (32.1)	0.25
2‐ Laser	27 (100.0)	63 (77.7)	0.006
3‐ Mechanical + Laser	12 (44.4)	15 (18.5)	0.008
Manual traction alone	0	7 (8.6)	
Femoral workstation	3 (11.1)	11 (13.6)	1.000
Fluoroscopy time (m) Median	50.2 (7.5–109.0)	15.7 (0.7–196.0)	< 0.001
Procedure time (m) Median	275 (72–625)	157.5 (45–460)	< 0.001
Procedural success	26 (96.3)	69 (85.2)	0.18
Clinical success	27 (100)	75 (92.6)	0.33
Failure	0	6 (7.4)	0.33
Major Complications	1 (3.7)	4 (4.94)	1.000
Minor Complications	3 (11.1)	3 (3.7)	0.16

* Counts reflect ICD leads removed; a patient may contribute > 1 explanted ICD lead (e.g., an abandoned/nonfunctional lead from prior revision).

Among the 27 IVL patients, complete IVL applications along the entire length of the leads from brachiocephalic vein to superior vena cava (SVC) were achieved in 20. In the remaining 7, applications were limited by proximal venous stenosis. IVL was not applied in the right atrium RA or right ventricle RV. Overall, the procedural and clinical success rates were not significantly different between the groups (Table 2).

Similarly, the rates of major and minor complications were not significantly different between the groups (Table 2). Only one case of vascular laceration with perforation requiring intervention was recorded in the IVL group. In this case, IVL was performed prior to attempted TLE of a biventricular device. The RA lead was the first lead attempted with good progress utilizing a 14Fr laser to the level of the SVC/RA junction, where dense fibrosis and lead‐to‐lead binding were noted. The operator decided to switch to the CS lead. Acute decompensation with development of tamponade occurred 15 min after the last laser application, during applying mild to moderate traction on the CS lead while advancing the tool onto that lead. In the conventional extraction groups, two patients developed pericardial effusion requiring intervention, and two other cases sustained vascular lacerations with tears requiring a sternotomy to control the bleed.

## 4 Discussion

Our cohort represents the largest reported series to date, with 27 IVL patients matched to 81 controls for comparative analysis. The IVL group exhibited longer fluoroscopy times and technical limitations but achieved comparable procedural success and safety outcomes.

Latanich et al. previously demonstrated that IVL could reduce active extraction time by 25 min in 14 complex TLE cases [3]. Theoretically, using IVL in cases with significant calcification would facilitate dissection of adhesions, potentially enabling extraction with manual traction alone. However, in our series IVL cases required more frequent use of powered sheaths, underscoring the underlying procedural complexity and incomplete adhesion modification despite adjunctive IVL. Nonetheless, the absence of procedural failure in the IVL cohort suggests a possible clinical utility that may become evident in larger studies. Although we did not record lead active extraction time, the longer fluoroscopy and procedural durations likely reflect the added steps required for balloon delivery and pulse application.

Importantly, success and complication rates in our series remained comparable to controls, consistent with the safety profile observed by Latanich et al. [3]. The unexpectedly higher complication and lower success rates in our control group compared to historically expected rates may in part be accounted by longer dwell times to match the IVL group. Nevertheless, the vascular laceration case in the IVL group highlights that while IVL may aid extraction, procedural risks remain. Larger studies are needed to better evaluate how pressure waves and extraction tools may contribute to excessive strain on the venous wall while extraction, increasing the overall risk of vascular lacerations.

Recently, Qin et al. described three cases of complex TLE in which IVL enabled successful removal after failed attempts with conventional tool [4]. In our cohort, IVL was employed preemptively before conventional extraction, reflecting its planned use as an adjunct rather than a rescue strategy.

We also observed technical limitations: one‐quarter of patients could not receive full IVL therapy due to proximal venous stenosis, a detail not emphasized in prior reports. This limitation may affect patient selection and procedural planning.

Operator experience with TLE after IVL varied from case to case. In some instances, the degree of fibrosis and binding seemed much less than expected suggesting a benefit from pretreating with IVL. In others, fibrosis and binding remained quite dense despite pre‐treatment. This highlights the challenges of small, observational studies, as well as technical challenges of this early experience as application was prevented in areas of occlusion and may not have been optimal in areas of poor balloon‐to‐lesion apposition, such as in large SVCs or in the RA and RV. Additionally, control patients were not matched by operator which may introduce bias when interpreting TLE outcomes.

## 5 Conclusion

In summary, in this cohort we did not observe a clear clinical benefit, underscoring the need for larger prospective studies to draw significant clinical conclusions and investigate the optimal use and patient selection.

## Funding

The authors received no specific funding for this work.

## Ethics Statement

The authors have nothing to report.

## Conflicts of Interest

The authors declare no conflicts of interest.

## Data Availability

The data that support the findings of this study are available from the corresponding author upon reasonable request.
